# Lupus nephritis pathology prediction with clinical indices

**DOI:** 10.1038/s41598-018-28611-7

**Published:** 2018-07-06

**Authors:** Youzhou Tang, Weiru Zhang, Minfeng Zhu, Li Zheng, Lingli Xie, Zhijiang Yao, Hao Zhang, Dongsheng Cao, Ben Lu

**Affiliations:** 1 Nephropathy & Rheumatology Department, 3rd Xiangya Hospital, Central South University, Changsha, Hunan China; 2 Department of Rheumatology and Immunology, Xiangya Hospital, Central South University, Changsha, Hunan China; 3 School of Pharmaceutical Sciences, Central South University, Changsha, China; 4 Hematology Department, 3rd Xiangya Hospital, Central South University, Changsha, Hunan China

## Abstract

Effective treatment of lupus nephritis and assessment of patient prognosis depend on accurate pathological classification and careful use of acute and chronic pathological indices. Renal biopsy can provide most reliable predicting power. However, clinicians still need auxiliary tools under certain circumstances. Comprehensive statistical analysis of clinical indices may be an effective support and supplementation for biopsy. In this study, 173 patients with lupus nephritis were classified based on histology and scored on acute and chronic indices. These results were compared against machine learning predictions involving multilinear regression and random forest analysis. For three class random forest analysis, total classification accuracy was 51.3% (class II 53.7%, class III&IV 56.2%, class V 40.1%). For two class random forest analysis, class II accuracy reached 56.2%; class III&IV 63.7%; class V 61%. Additionally, machine learning selected out corresponding important variables for each class prediction. Multiple linear regression predicted the index of chronic pathology (CI) (Q^2^ = 0.746, R^2^ = 0.771) and the acute index (AI) (Q^2^ = 0.516, R^2^ = 0.576), and each variable’s importance was calculated in AI and CI models. Evaluation of lupus nephritis by machine learning showed potential for assessment of lupus nephritis.

## Introduction

Systemic lupus erythematosus (SLE) is a multisystem autoimmune disease with unclear etiology, one of its most serious manifestations is lupus nephritis. Various factors contribute to lupus renal damage, including complement, autoantibodies, environment, and genetics^1,2^. Numerous clinical and laboratory parameters, such as a panel of urinary proteins and autoantibodies^3,4^, have been explored as indices for lupus nephritis severity assessment and prognosis prediction. However, proper assessment of lupus nephritis depends on comprehensive analysis of multiple parameters rather than a single one, yet clinicians do not always agree on which parameters were important, nor do they have effective ways to evaluate some controversial parameters.

Lupus nephritis assessment involves classifying disease pathology into six classes and renal pathology acute index (AI) and chronic index (CI). Defined by the International Society of Nephrology/Renal Pathology Society (ISN/RPS), lupus nephritis were classified as: class I, minimal mesangial; class II, mesangial proliferative; class III, focal; class IV, diffuse segmental or global; class V, membranous; and class VI, advanced sclerosing^5^. Each class has its unique pathogenic mechanism, hence the corresponding therapy varies according to the American College of Rheumatology guidelines^6^. Lupus nephritis assessment also involves evaluating the indices of acute and chronic renal lesions which have potential to predict renal prognosis^7^. Lupus nephritis patients with different AIs and CIs respond differently to standard therapy^8^. In addition, patients with higher AI and CI are more likely to have acute kidney injury, which directly affects renal outcomes^9^. Currently, only through renal biopsy could clinicians obtain classification and AI and CI results.

Although renal biopsy is the gold standard reference for lupus nephritis evaluation, it is an invasive operation. Therefore, a noninvasive method as a supplementation for biopsy is highly warranted. This noninvasive approach helps integrating numerous clinical parameters, providing a reliable understanding of lupus nephritis pathology features and prognosis and even select out important clinical indices. Machine learning based on mathematical algorithms is well suited to such a challenge. Actually machine learning has already been used to predict some multi-factors affected diseases and had promising results^10,11^. For lupus, an artificial intelligence-based approach to SLE prediction has shown potential, even though it only evaluated autoantibodies^4^. In advanced machine learning, the collected data would be divided into “training set” and “test set”: data in a so-called “training set” are selected and integrated into a prediction model; during this process, certain variables may be weighted more heavily than others. Then the prediction model is validated by data in itself or against a so-called “test set”. This data-based approach can be continuously revised as the available data base grows.

Here we developed and validated machine learning-based models for classifying the histology of lupus nephritis and predicting indices of acute (AI) and chronic (CI). Valuable parameters were selected out and weighed for their importance.

## Results

### Patient characteristics

Analysis of disease characteristics across 173 patients includes 43 males and 130 females with lupus nephritis by different pathological classes (Table 1). Treatments recommended by the American College of Rheumatology for patients with class I or II disease depend on whether an extra-renal condition is present, and different treatments are recommended for class III & IV than for pure class V. In addition, disease involving a combination of class V with class III or IV should be treated as pure class III or IV disease^6^. Therefore we defined three clusters of pathological classes: cluster 1 (class II), cluster 2 (class III or IV, including V combined with III or IV) and cluster 3 (pure class V). In this way, cluster 1 has 58 samples while cluster 2 includes 82, cluster 3 has 33. Random forest algorithm was generated for these three clusters.

**Table 1:** Patient characteristics, stratified by histological classification.

Characteristic	Class			Total (n = 173)
	**II (n = 58)**	**III and IV (n = 82)**	**V (n = 33)**	
Mean age, yr	27.49 ± 13.26	26.38 ± 13.65	28.27 ± 13.83	27.12 ± 13.5
Female	84.7^#^	67.9	75.8	75.1
Fever	23.7	25.9	24.2	24.9
Photosensitivity	18.6	19.8	30.3	21.4
Psilosis	20.3	25.9	9.1	20.8
High blood pressure	11.8^*^	29.6^*^	21.2	22
Arthralgia	47.5	33.3	33.3	38.2
Edema	44.1	46.9	48.5	46.2
Erythra	35.6^*^	52.9	51.6	46.9
OB (+)	54.2	63	54.5	58.4
Raynaud phenomenon	6.8	11.1	9.1	9.2
Urinary protein(+)	83.1	85.2	87.9	85
Urinary erythrocytes(+)	35.6	54.3	51.5	47.4
WBC count (10^9^/L)	7.59 ± 3.89	6.86 ± 4.02	7.94 ± 7.56	7.31 ± 4.85
PLT count (10^9^/L)	219.36 ± 108.22^*#^	167.63 ± 74.55^*^	178.53 ± 86.1	187.35 ± 92
BUN (mmol/L)	6.79 ± 5.1	8.73 ± 7.12	7.84 ± 5.49	7.9 ± 6.22
Cr (μmol/L)	104.28 ± 132.81	111.54 ± 107.96	93.14 ± 61.02	105.55 ± 110.04
Uric acid (μmol/L)	335.31 ± 112.94^*#^	411.03 ± 125.4^*^	364.12 ± 118.7	376.26 ± 133.77
Serum C3 (10^3^ g/l)	691.19 ± 253.42^*^	507.47 ± 301.17^*#^	755.85 ± 346.2^*※^	617.5 ± 312.05
SSB(+)	11.8	8.6	12.1	10.4
SLEDAI	12.71 ± 5.63	14.81 ± 6.71^*^	12.61 ± 5.62	13.68 ± 6.22
TIL	3.05 ± 1.33^*#^	3.81 ± 1.68	3.94 ± 1.64	3.58 ± 1.6
NAG (U/L)	12.7 ± 3.91^*#^	16.33 ± 1.77	17.55 ± 3.01*	15.33 ± 3.77
AI	4.8 ± 2.93	7.28 ± 2.62	5.73 ± 3.16	6.13 ± 3.04
CI	0.9 ± 1.16	1.74 ± 2.09	1.79 ± 1.87	1.46 ± 1.82
eGFR (ml/min)	105.49 ± 66.05	102.52 ± 73.92	98.21 ± 45.35	102.71 ± 66.35
Serum C4 (10^3^ g/l)	145.1 ± 71.45^#^	118.03 ± 74.14^*^	136.32 ± 72.34	130.75 ± 73.51
Sm(+)	16.9	24.7	21.2	21.3
SSA(+)	49.2	44.4	51.5	47.4
nRNP(+)	15.3	18.5	6.1	15
dsDNA(+)	22^*#^	56.8^*※^	18.2^*^	37.6
Scl-70(+)	5.1	1.2	0	2.3
Jo-1(+)	3.4	1.2	0	1.7

The data included 173 samples. According to treatment differences, we defined three clusters of pathological classes: cluster 1 (class II), cluster 2 (class III or IV, including V combined with III or IV) and cluster 3 (pure class V). Serum C3: serum complement 3, Serum c4: serum complement 4, dsDNA: double-stranded DNA, SLEDAI: systemic lupus erythematosus Disease Activity Index, Cr: creatinine, SSB: anti-Sjogren syndrome B antibody, eGFR: estimated glomerular filtration rate, nRNP: U1-RNP antibody, OB: stool occult blood, WBC count: white blood cell count, PLT count: platelet count, TIL: tubulointerstitial lesion, NAG: urinary N-acetyl-beta-d-glucosaminidase isoenzyme, AI: renal biopsy acute index, CI: renal biopsy chronic index. Values are expressed by % or mean ± SD. *p < 0.05, comparing to the other two groups, ^#^*p* < 0.05, class II vs class III&IV, ※*p* < 0.05, class III&IV vs class V, *p < 0.05, class II vs class V (chi-square test was used for classified variables, ANOVA test was used for continuous variables).

According to direct comparisons, we found some interesting phenomena as listed in Table 1: first, class III&IV bear higher blood pressure and higher levels of uric acid, which is in accordance with clinical observations that class III&IV patients have more severe and accelerated renal damage; second, complement 3 (C3) and complement 4 (C4) levels are lower in Class III&IV patients, which implies higher complement mediated immune damage; third, class III&IV have higher tubulointerstitial lesion (TIL) score and urinary N-acetyl-beta-d-glucosaminidase isoenzyme (NAG) enzyme level which reflects more severe tubular and interstitial damage; additionally, some differences exist between classes like double-stranded DNA (dsDNA), systemic lupus erythematosus disease activity index (SLEDAI), erythra, etc. However, analysis with only single variable is not as accurate as comprehensive researches based on multiple variables together to evaluate lupus nephritis; selecting out valuable variables accurately is still uneasy. Machine learning applied in our research could be regarded as a suitable way.

### Prediction of pathologic classification

**Figure 1:**
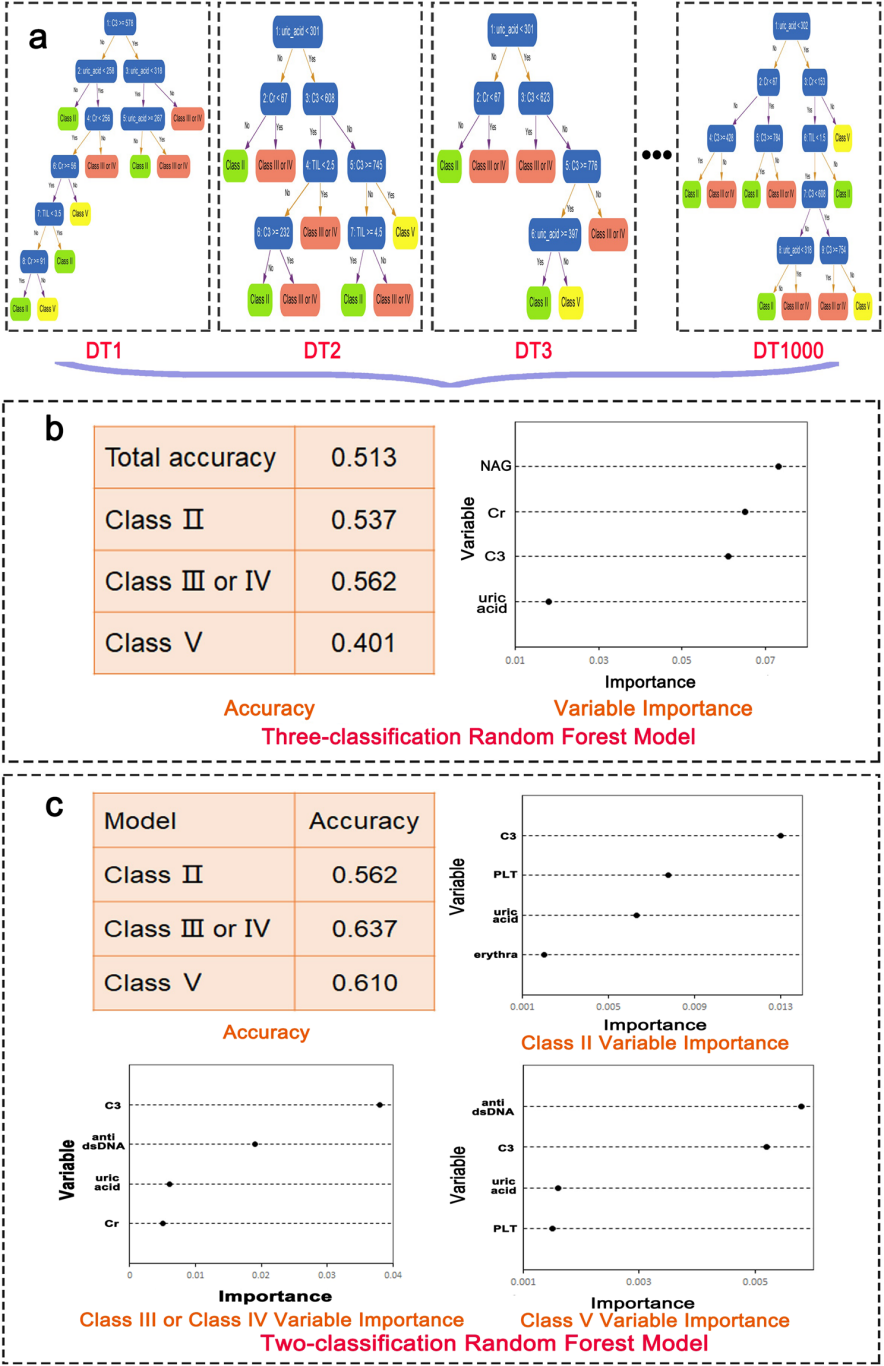
Random forest algorithms for class prediction and variable selection. (**a**) Random forest schematic diagram showing how it worked. The forest consists of 1000 decision trees. In each tree, samples were classified into two “leaf nodes” by a randomly chosen variable, continue the dividing process using different variables randomly until all the samples were classified into three clusters. (**b**) The table showed three classification random forest prediction accuracy results on total and each cluster of classes; The right diagram showed the prediction model importance of four main variables: urinary NAG enzyme, Cr, serum C3 levels and uric acid level. (**c**) Two-classification random forest prediction accuracy were listed in the table. Each class has its unique panel of specific variables, different variables’ importance were shown in diagrams.

Random forest belongs to high accurate algorithms and runs efficiently on large data treatment. Because of its high efficiency in classification prediction, we applied random forest to predict LN classes. Figure 1a illustrated the schematic diagram how random forest worked to classify patient samples depending on variables. In the diagram, the forest consists of 1000 decision trees which were the basic unit for the “forest”. For each single tree, machine randomly chose a variable to divide samples into different “leaf nodes”, continue this process by randomly choosing different variables until all samples were classified into concrete clusters (classes). In this way, a decision tree was generated. Learning from a certain number of trees, the forest summarized its classification rule depending on vital parameters and bore competence to predict samples into classes. Random forest generated for the three clusters of pathologic classes at the same time showed a total accuracy of 51.3%, 53.7% for class II, 56.2% for class III&IV and 40.1% for class V (Fig. 1b). The system picked out some variables as important for prediction, based on their assigned weights, these include urinary NAG enzyme level, creatinine (Cr), C3 and uric acid (Fig. 1b). These give us clues that these variables were important characters to differentiate classes among total variables, which is in accordance with our previous direct comparison shown in Table 1.

The above algorithms used random forest to classify three clusters at the same time and provided some clues. The results are better than random classification (33.3%), however, there is room for improvement. To further explore prediction ways for each cluster and investigate each cluster’s distinguishing characters compared to the others, we used random forest generated for two classes. In this calculation, random forest divided samples into 2 clusters at the same time, class II vs none class II, class III&IV vs none class III&IV, class V vs none class V. In this way, class II accuracy reached 56.2%, class III&IV 63.7% while class V reached 61% which we could see an improvement especially for class V (Fig. 1c). For the two-classification, random forest found C3, platelet (PLT) count, uric acid and erythra were vital variables for class II, C3, dsDNA antibody, uric acid and Cr as important to feature class III&IV while dsDNA antibody, C3, uric acid and PLT were included in class V prediction (Fig. 1c). These results were very meaningful by depicting each cluster’s character. Among them, uric acid and C3 were sensitive variables implying different levels of early renal lesion and complement related immune damage. Additionally, based on two-class random forest models, dsDNA is another important character, lower erythra rate is a vital feature for class II.

### Prediction of AI and CI

**Figure 2:**
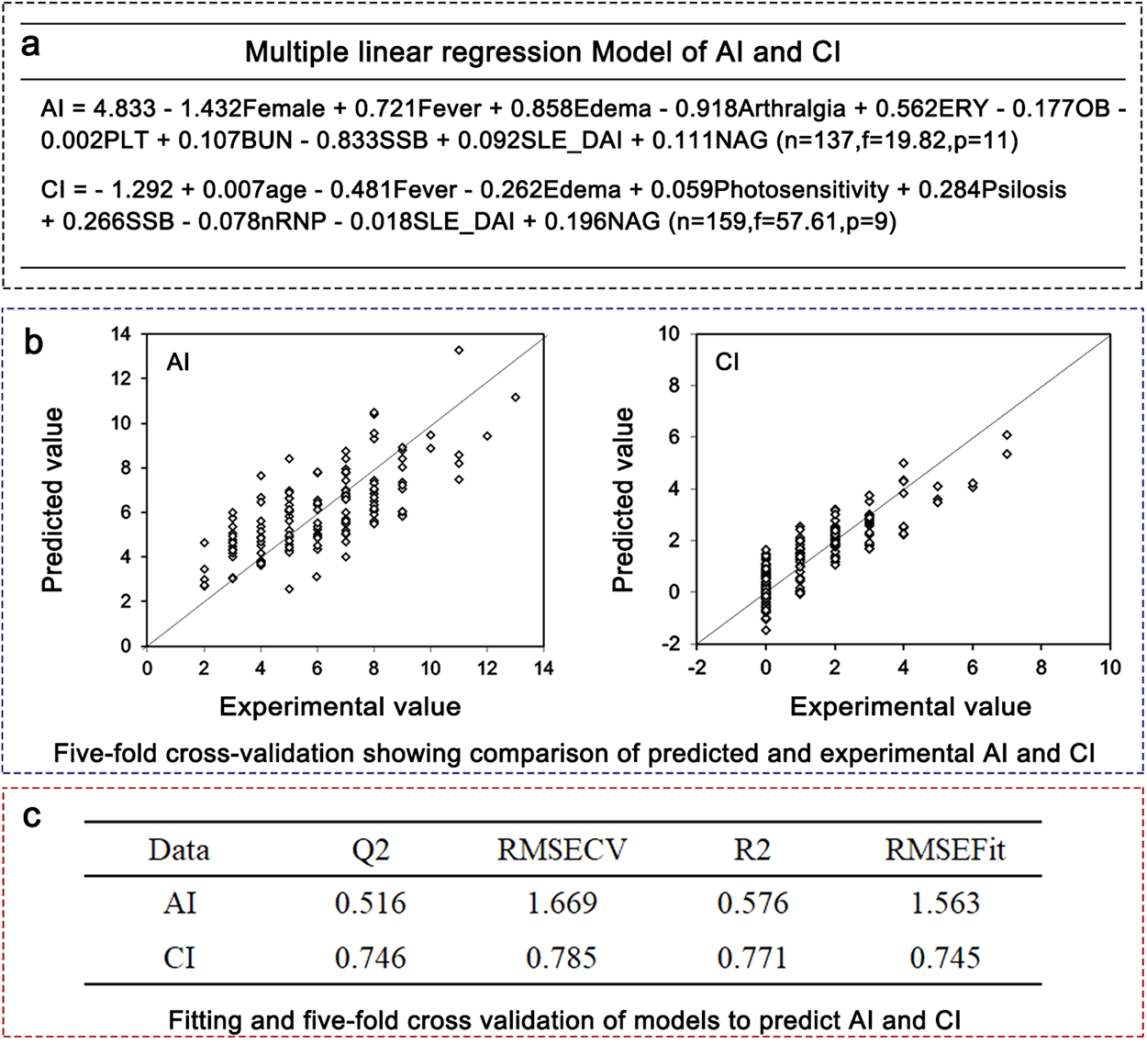
Multiple linear regression algorithms to prediction AI and CI. (**a**) Multiple linear regression models for AI and CI. Using this statistical method, multiple variables were integrated and constructed equations for AI and CI. In multiple linear regression models of AI and CI, sex: 1 (male) or 2 (female); fever: 0 (absent) or 1 (present) (likewise for edema, arthralgia, OB, SSB, and U1-RNP (nRNP); and urinary erythrocytes (ERY): 0 (none), 1 (+), 2 (++), 3 (+++). platelet (PLT) count (10^9^/L), urinary NAG enzyme level (U/L) and BUN (mmol/L) were treated as continuous variables. For each equation, n (sample numbers), f score and p (variable numbers) were listed. (**b**) Validations on models: Diagrams showed prediction models’ fitness to biopsy pathological results using five-fold cross validation; (**c**) Fitting and five-fold cross validation table to predict AI and CI showed satisfying results on models for CI (Q^2^ = 0.746, R^2^ = 0.771) and AI (Q^2^ = 0.516, R^2^ = 0.576). Q^2^ reflect test set validation result and R^2^ reflected validation on itself (divide model data into 5 even part, used 1/5 to test models).

Besides classification, we applied machine learning on acute and chronic indices prediction. Multilinear regression were used to develop models. Based on existing variables, two equations were built to obtain AI and CI scores (Fig. 2a). To further validate the models’ accuracy, fitting and five-fold cross validation were used (Fig. 2b,c). This method showed satisfactory results on models for CI (Q^2^ = 0.746, R^2^ = 0.771) and AI (Q^2^ = 0.516, R^2^ = 0.576). Q^2^ represents prediction fitness of training set to test set and R^2^ represents model prediction validation on itself.

**Figure 3:**
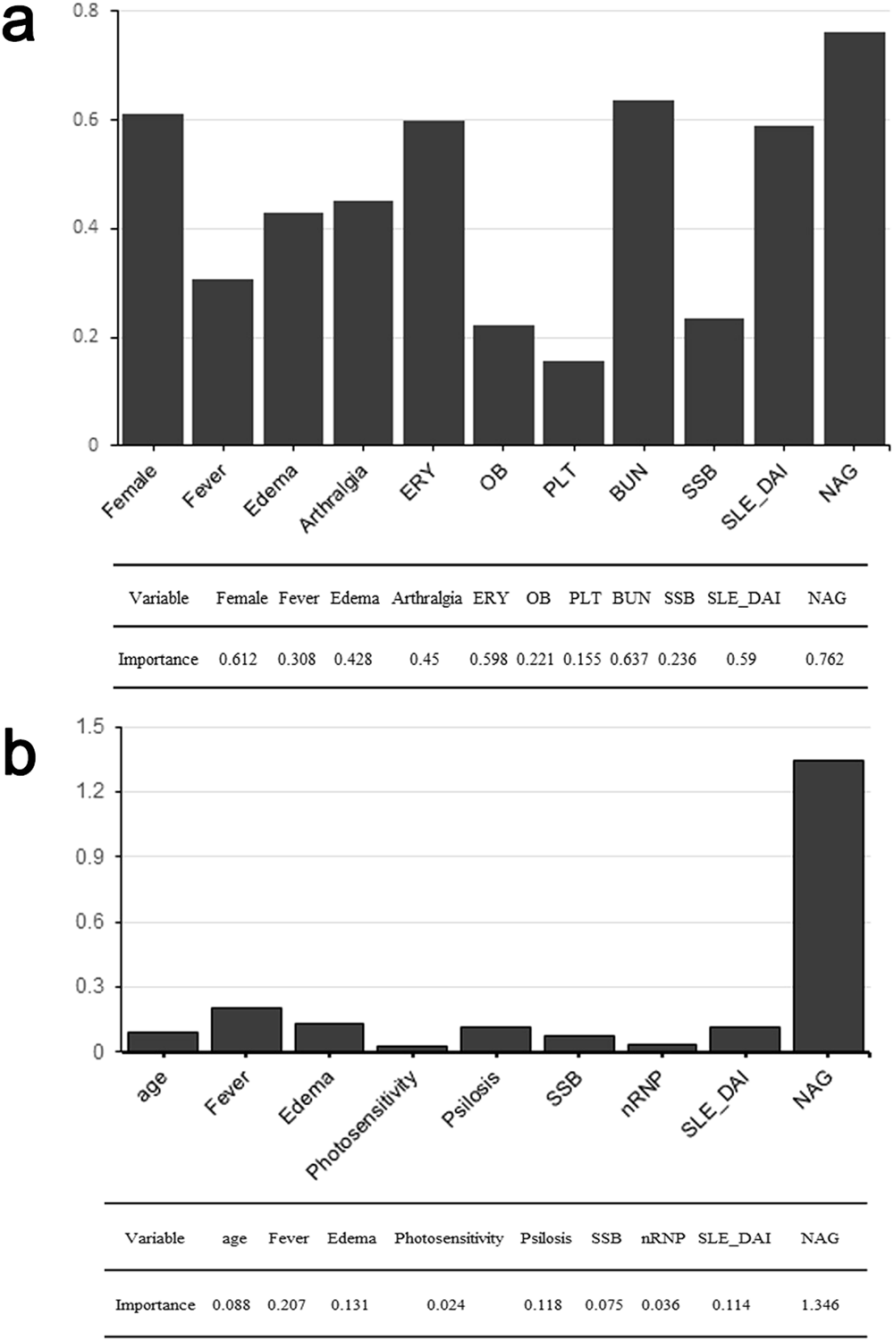
Variable importance in AI and CI prediction. Variable importance for AI (**a**) and CI (**b**). Based on Fig. 2 models for AI and CI prediction, the diagrams shows each variable’s influence on models. We used vector cosine similarity to evaluate SLEDAI’s relation with other variables in equations and it showed that SLEDAI is comparably an independent variable in AI and CI equations.

Next, to further investigate each variable’s contribution to models, machine learning evaluated weight of model variables. It found importance and found some indices such as urinary NAG enzyme level, sex, arthralgia, BUN, edema, fever, SLEDAI and anti-Sjogren syndrome B antibody (SSB) were important variables for AI prediction (Fig. 3a); while urinary NAG enzyme level, age, fever, psilosis, edema, SSB and SLEDAI were important for CI prediction (Fig. 3b). To further investigate whether SLEDAI was an independent variable in equations, vector cosine similarity was used to evaluate SLEDAI’s relation with other variables. It showed that SLEDAI is comparably an independent variable in AI and CI equations. However, although some variables such as urinary NAG level devoted much in models, machine could not generate satisfying prediction results based on any single variable, only comprehensive analysis based on multiple variables gives satisfactory outcomes.

## Discussion

Here we used a machine learning-based approach to predict pathologic classes as well as AI and CI of lupus nephritis. On the whole, this method showed prediction competence and provided multiple clues for lupus nephritis.

This artificial intelligence is novel and has its unique advantages: it’s noninvasive; it integrates multiple irrelevant variables simultaneously and reasonably evaluated the weight of each variables; it can be refined continuously as database enlarges and sensitive variables are constantly been discovered. As a developing technology, it is convenient and suitable to be a clinical tool for continuous disease monitoring.

However, artificial intelligence still has some room for improvements, especially in prediction accuracy instability. The three class random forest efficiency were not as satisfactory as we expected, especially for class V. This may reflect, in part, the uneven sample distribution. However, we speculate that it reflects the disease complexity. As we know, lupus nephritis etiology is obscure and relates to various known and unknown reasons, the pathological lesions vary as immune damage mechanisms vary. For instance, some researchers suppose class IV lupus nephritis has predominant Th1 bias in peripheral T helper cells^12^. Indeed, previous studies have proposed a range of inflammation mechanisms-based parameters including relative amounts of Th1 and Th2 cytokines, relative amounts of IgG3 or IgG4 autoantibodies^12–14^, urinary levels of proinflammatory factor HMGB1^15^, serum levels of renalase^16^ and levels of urinary podocyte mRNA^17^. However, the above parameters are not used routinely. Therefore, improving class prediction may require deeper exploration of different inflammatory mechanisms and their sensitive parameters.

It is worth mentioning that our methods recognized some valuable parameters especially that we first concluded different panels of specific variables to character each class by two-class random forest.

For clinical symptoms, erythra and arthralgia gave interesting results:

Class II bears lower erythra rate and erythra was perceived as a valuable parameter for class II by random forest. We suppose that lupus nephritis lesion severity is in some degree associated with dermal lesion. For further investigation, we will divide erythra into different subgroups as butterfly erythema/discoid rashes, rashes in face/body and analyse their relation with lupus nephritis.

By our algorithms, arthralgia contributes to AI prediction. Arthralgia in SLE is usually acute and wandering, so it may be part of the acute pathology of lupus nephritis. Additionally, research on synovial fluid samples may suggest new indices worth investigating.

Lupus nephritis damage includes not only glomerular, but also interstitium, tubular and vessels. TIL has been found to be important risk factor for renal outcomes in lupus nephritis. When calculating TIL indices, tubular cell degeneration and necrosis, interstitial inflammatory cell filtration, interstitial fibrosis and tubular atrophy should be considered^18,19^. It was scored by the above aspects. As a pathological index, TIL is an important independent factor for lupus nephritis prognosis^20^. Alleviate TIL is very important for long term renal function protection. We used urinary NAG enzyme which was considered a sensitive index reflecting TIL condition^21,22^. Our data showed that urinary NAG differs between classes, which is consistant with the observation that secondary tubulointerstitial nephritis co-occurs with lupus glomerulonephritis and is more frequent in class IV lupus nephritis^23^. In AI and CI models, we used urinary NAG enzyme level to reflect tubulointerstitial condition. It devoted to equations especially for CI (remove NAG enzyme from AI and CI equation, the prediction accuracy dropped obviously). This phenomenon gives us clues. Currently, lupus nephritis classes were defined mainly by different glomerular changes. Glomerular lesion is the main aspect in acute and chronic renal damage. Our results indicates that tubular and interstitial lesion level was vital to lupus nephritis classification, therefore playing an important role in acute and chronic renal damage. Based on current technology, machine learning has potential to be developed as a satisfactory tool but could not replace renal biopsy. Nevertheless, as we mentioned, our trial is the first step, as a helper tool, machine learning gives us clues for indices selection, artificial intelligence has ability to learn from continuously expanded database and novel explored indices. It selected out NAG as vital index, we could try other ways to explore more sensitive clinical indices reflecting tubular interstitial lesion level.

Ours is the first report, to our knowledge, to links SSB autoantibody to AI and CI. SSB antibody is auto-antibody against La protein which recognizes small RNA and affects RNA activity and stabilization. SSB antibody exists only in auto-immune diseases patients as SLE, Sjogren’s syndrome. This antibody relates to dsDNA^24^ and gives clues to SLE prognosis, therapeutic guidance, patients with lupus nephritis appear to suffer less if they possess La autoantibodies together with Ro antibody^25,26^. As we have already found SSB antibody is a valuable parameters for AI and CI prediction, further study is needed to accurately assess the titer of these antibodies to the pathogenesis of lupus nephritis and the relationship between SSB and TIL, dsDNA, C3, etc. Additionally, some other indices as uric acid has previously been reported to be a sensitive variable in lupus nephritis development which correlates negatively with C3 levels^27^. In our models, it is also one of the important variables selected out to predict classes.

Machine learning is an attractive prediction method and our preliminary trials give us valuable clues for deeper exploration. Further researches needed to continue exploring novel and sensitive parameters; calculate continuous variables’ specific range for each class such as uric acid, serum C3 level, TIL, etc.; refine and specify variables as TIL, erythra and observe their relationship with lupus nephritis. With the enlargement of data, machine learning will gain power and become a promising approach in lupus nephritis prediction.

## Material and Methods

### Patients

This study involved 173 inpatients who were treated at nephropathy departments in Xiangya Hospital and The 3^rd^ Xiangya Hospital of Central South University (Changsha, China) from Jan 2014 to Jan 2016. All patients were informed of the purposes of the study and signed written consent before enrollment. This research had no manipulation on patients’ treatment, according to policy of Central South University, ethics board approval was not required.

### Collection of clinical and pathology data

Data were retrospectively collected from each patient’s medical records on clinical symptoms and laboratory results. We collected information in three areas: general information including age and sex; clinical symptoms including fever, hypertension, erythra, edema, arthralgia, photosensitivity, Raynaud’s phenomenon and psilosis; laboratory results including routine urine test (proteinuria, hematuria), stool route (occult blood test (OB)), blood route (white blood cell (WBC), platelet (PLT)), renal function test, serum complement (complement 3(C3), complement 4 (C4)), auto-antibody tests, SLE Disease Activity Index (SLEDAI), tubulointerstitial lesion (TIL)^22,23^ (score on 4 aspects: tubular cell degeneration and necrosis, tubular atrophy, interstitial inflammation cell infiltration and interstitial fibrosis. None with 0, mild with 1, moderate with 2, severe with 3) and estimated Glomerular Filtration Rate (eGFR) calculated according to Chronic Kidney Disease Epidemiology Collaboration (CKD-EPI) equation^28^. Data collectors were two medical doctors who were blind to patients’ pathological reports.

For each patient, an experienced clinician calculated SLEDAI, and an experienced pathologist analyzed renal biopsy pathology results to classify the histology type of lupus nephritis as well as calculate indices on TIL, AI and CI.

According to International Society of Nephrology/Renal Pathology Society’s definition^5^, lupus nephritis class I mininal mesangial represents for lupus nephritis with none or very mild renal pathology changes; class II mesangial proliferative presents mesangial hypercellularity or matrix expansion; class III focal involves less than 50% glomeruli with active or chronic or active and chronic lesions on endo- or extra-capillary regions; class IV diffuse involves more than 50% glomeruli (diffuse) lesions, either segmental or global; class V membranous has global or segmental subepithelial immune deposits causing membranous changes, sometimes it combines with class III or class IV pathology; class VI advanced sclerosing’s main change is global sclerosis with more than 90% glomeruli.

### Statistical analysis

To analyze data in Table 1, we used chi-square test for classified variables, ANOVA test for continuous variables.

In Fig. 1, we used “Random Forest” which belongs to “Ensemble Learning” applied to classification predictions. Random forest has the potential to accommodate a large number of independent variables. First, data was be divided into training set and test set. Training set was used to build the model. A single “decision tree” which is the basic unit of the “forest” represents the classification procedure using multiple independent variables in random order. Depending on different variables, the patients would be classed into subgroups (leaf nodes), this procedure would continue until all the patient samples were classified. This forms a decision tree. In our model, we used 1000 trees to build the forest. Then to validate the model, a test set was be used to run the process. Not only that, random forest estimate each variable’s importance in the classification model: first count the number of patients correctly classified, then change the value of one independent variable into the opposite value in each tree (e.g., change the age value or change auto-antibody from positive to negative), run the tree procedure again, the relative importance is defined by the ratio of the number of correct classified patients after the alteration to the number of correctly classified patients before alteration.

To predict pathology indices, we used 137 patients’ data in the case of AI and 159 in the case of CI. Multilinear regression was used to build these prediction models. After model fitting and five-fold validation, each variable was analyzed by stepwise regression to determine its importance (weight) to influence prediction models.

All statistical methods used above were instructed by R Language 3.3.2 (http://cloud.r-project.org/bin/windows/base/R-3.3.2-win.exe). The random forest models were built by random forest package from CRAN (https://cran.r-project.org/web/packages/); classification variables’ importance were calculated by mean decrease accuracy method in random forest package. The multiple linear regression models were built by multiple linear regression model method in R language.
